# Sleep Disturbance and Its Clinical Implication in Patients with Adult Spinal Deformity: Comparison with Lumbar Spinal Stenosis

**DOI:** 10.1155/2020/6294151

**Published:** 2020-04-13

**Authors:** Ho-Joong Kim, Seok-Jun Hong, Joon-Hee Park, Hojoon Ki

**Affiliations:** ^1^Spine Center and Department of Orthopaedic Surgery, Seoul National University College of Medicine and Seoul National University Bundang Hospital, 166 Gumi-ro, Bundang-gu, Sungnam 13620, Republic of Korea; ^2^Department of Anesthesiology & Pain Medicine, Kangdong Sacred Heart Hospital, Hallym University College of Medicine, Seoul 134-701, Republic of Korea

## Abstract

**Purpose:**

The purpose of this study was to investigate the prevalence of sleep disturbance and its clinical implication in patients with ASD.

**Methods:**

A total of 44 patients with ASD and 137 patients with lumbar spinal stenosis (LSS) were enrolled in the study. Forty four patients were selected from the LSS group after propensity score matching. Global Pittsburgh Sleep Quality Index (PSQI) score, demographic data, visual analog scale (VAS) score for back and leg pain, Oswestry Disability Index (ODI), and EuroQol 5-dimension questionnaire (EQ-5D) were compared between both groups. Multiple regression analysis was performed with VAS for back pain as the dependent variable and age, sex, PSQI, and VAS for leg pain as the independent variables in the ASD group.

**Results:**

33 (75.0%) and 32 (72.7%) patients were classified as poor sleepers in the ASD group and the LSS group, respectively. In the ASD group, the VAS score for back pain was 7.7 ± 1.7 in the poor sleeper group and 5.6 ± 2.2 in the nonpoor sleeper group. In the LSS group, poor sleep quality was associated with the ODI score, ODI score without a sleep component, and EQ-5D. The regression model for predicting VAS for back pain in the ASD group suggested that poor sleep quality and increased leg pain were associated with increased back pain.

**Conclusions:**

Because sleep quality is a critical factor in augmenting back pain in patients with ASD, this study underlines the need to investigate sleep quality during the routine examination of patients with ASD.

## 1. Introduction

Sleep disturbance is a common problem among adults, with a prevalence of 27.0–38.3% in adults aged 50–70 years in South Korea [1–3]. Poor sleep quality can have adverse effects on pain, quality of life, various morbidities, and health-care use [4]. In addition, chronic medical conditions are important contributors to sleep difficulties and, more specifically, it has been reported that there is an association between painful musculoskeletal disease and sleep problems [3, 5–10].

Previous studies have shown that degenerative spinal disease is related to a poor sleep quality, which leads to increased pain severity and disability, poor quality of life, and adverse mental health outcomes [6, 7, 11]. Approximately, 80% of patients with chronic low back pain have a poor sleep quality [6, 7]. Ohtori et al. reported that nocturnal leg cramps frequently occur in patients with lumbar spinal stenosis (LSS) [12]. Thus far, however, there have been no studies on the prevalence of poor sleep quality in patients with specific degenerative spine diseases. Only one recent study has shown that sleep disturbance is very common in patients with LSS and is correlated with symptom severity [13]. Therefore, an intervention for improving sleep quality would aid in improving the quality of life and minimizing disability in patients with degenerative spinal disease.

Adult spinal deformity (ASD) is predominantly a problem of old age, with a prevalence of up to 32% in patients aged >50 years and 68% in patients aged >70 years; thus, ASD has been the subject of recent rigorous investigations [14–17]. However, there has been no study about the relationship between ASD and sleep disturbance. Thus, the purpose of this study was to investigate the prevalence of sleep disturbance and its clinical implication in patients with ASD.

## 2. Materials and Methods

### 2.1. Study Design and Patients

**Figure 1:**
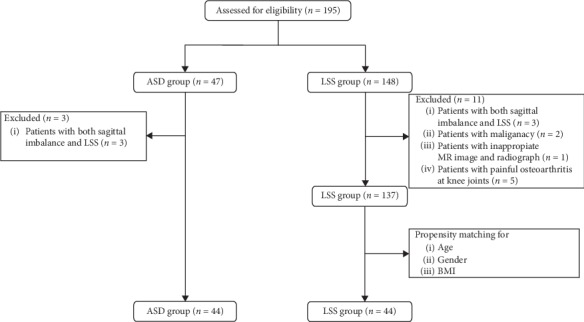
Flow diagram of enrollment, randomization, and follow-up of the study participants.

This cross-sectional study was approved by the institutional review board of our hospital, and all participants provided written informed consent before enrollment. A total of 195 consecutive patients who were scheduled to undergo spine surgery for ASD and LSS between January 2018 and October 2018 were assessed for eligibility in this study (Figure 1). The patients were categorized into an ASD group, comprising patients with ASD with kyphosis, and a control LSS group, comprising patients with LSS. All patients were scheduled to undergo spine surgery. The inclusion criteria for the ASD group were (1) age from 65 to 85 years and (2) a diagnosis of ASD with a positive sagittal balance and planned corrective surgery for ASD, defined as sagittal vertical axis >5 cm, pelvic tilt >20°, or pelvic incidence minus lumbar lordosis >20 on lateral radiographs in the standing position. The inclusion criteria for the LSS group were (1) age from 65 to 85 years and (2) presence of LSS diagnosed on the basis of the presence of a stenotic lesion on lumbar spine magnetic resonance imaging; corresponding neurogenic intermittent claudication; and 1 or more corresponding symptoms including pain, numbness, neurological deficits in the legs and buttocks, and/or bladder/bowel dysfunction [18, 19]. The exclusion criteria for both groups were as follows: (1) presence of both ASD and LSS; (2) severe pain due to any other musculoskeletal disease; (3) peripheral vascular disease; (4) inappropriate radiographs; (5) any syndromic, neuromuscular disease; (6) any serious uncontrolled medical comorbidity such as sepsis or malignancy that would cause disability or worsen general health; and (7) inability to complete the questionnaires on health-related quality of life (HRQOL) and disability.

According to the above criteria, finally 44 patients with ASD and 137 patients with LSS were enrolled in the study. To match with the ASD group, 44 patients were selected from the LSS group after propensity score matching for age, sex, and body mass index (Figure 1).

### 2.2. Assessment of Sleep Quality

Sleep quality was assessed using the Pittsburgh Sleep Quality Index (PSQI), an instrument with known reliability and validity [20]. The PSQI questionnaire consists of 19 self-report questions, each having an ordinal grading scale ranging from 0 to 3, in which 0 represents no current issues and 3 reflects the worst quality of sleep. These 19 questions are further divided into 7 subjective components that include sleep quality, sleep latency, sleep duration, habitual sleep efficiency, sleep disturbance, use of sleeping medications, and daytime dysfunction. The 7 components are summed to yield a global PSQI score, ranging from 0 to 21, with higher scores indicating poorer sleep quality. The index tool (PSQI) has acceptable internal consistency with a Cronbach reliability coefficient of 0.84 and has been validated in the Korean language [21]. A global sum of >5 indicates a poor sleeper based on a sensitivity of 0.90, specificity of 0.87, and a *k* value of 0.75 in distinguishing sleep quality [20]. This sum was used to dichotomize the enrolled patients into either the poor sleeper group or the nonpoor sleeper group.

### 2.3. Demographic Data and Clinical Evaluation

In addition to the PSQI, the following baseline clinical and demographic variables were collected: sex, age, height, weight, body mass index, medical history, LSS treatment, and clinical outcomes including visual analogue scale (VAS) scores for back and leg pain, the Oswestry Disability Index (ODI), and the EuroQol 5-dimension questionnaire (EQ-5D) [22–24].

ODI (2.0) is a self-administered questionnaire measuring back-specific function [22]. The questionnaire consists of 10 items, each with 6 response levels. Each item is scored from 0 to 5, and the total score is converted to a 0–100 scale. In addition, the ODI score without a sleep component was also calculated for analyzing the true association between poor sleep quality and disability.

The HRQOL was measured using the EQ-5D, a 5-dimensional health state classification. The 5 dimensions are mobility, self-care, usual activities, pain/discomfort, and anxiety/depression. An EQ-5D health state is defined by selecting 1 level from each dimension. The EQ-5D preference-based measure can be considered a continuous outcome scored on a 0–1.00 scale, with 1.00 indicating full health and 0 representing death [24].

### 2.4. Statistical Analysis

To compare the prevalence of sleep disturbance between the ASD and LSS groups, we performed propensity score matching. Logistic regression analysis was conducted to estimate the propensity scores of the patients in both groups. Independent *t*-tests and chi-square tests were used to analyze the differences between the ASD and LSS groups, as well as between the poor sleeper and nonpoor sleeper groups. For adjustment of confounding biases including age and sex, analysis of covariance was also conducted. Multiple regression analysis was performed with VAS for back pain as the dependent variable and age, sex, PSQI, and VAS for leg pain as the independent variables in the ASD group. The adjusted *r*^2^ values of the model were reported after regression analysis. The alpha significance level was set at 0.05. All statistical analyses were performed using SPSS version 20.0.0 software (SPSS Inc., Chicago, IL, USA).

## 3. Results

### 3.1. Descriptive Statistics

Table 1 demonstrates the baseline characteristics of the patients in the ASD and LSS groups. The demographic data were similar between the groups. The mean age ± standard deviation was 69.5 ± 7.7 and 69.9 ± 8.0 years in the ASD group and the LSS group, respectively. Each group had 10 male patients and 34 female patients. There were no clinical differences in clinical outcomes including back pain, leg pain, ODI, and EQ-5D between the 2 groups (Table 1). In addition, the global PSQI was not different between the 2 groups (*P*=0.375), and 33 (75.0%) and 32 (72.7%) patients were classified as poor sleepers in the ASD group and the LSS group, respectively. Among the subcomponents of the PSQI, in particular, the use of sleep medications had a significant negative impact on sleep interruption in LSS patients (*P*=0.034) than in ASD patients. No significant differences were found between ASD and LSS groups in regard to sleep quality, sleep latency, sleep duration, habitual sleep efficiency, sleep disturbance, or daytime dysfunction as contributors to the PSQI (Table 2).

### 3.2. Difference of Clinical Outcomes between Poor and Nonpoor Sleepers in Each Group

In the ASD group, poor sleepers had a significantly higher intensity of back pain than nonpoor sleepers. The VAS score for back pain was 7.7 ± 1.7 in the poor sleeper group and 5.6 ± 2.2 in the nonpoor sleeper group (*P*=0002; *P*=0.004 after adjustment for age and sex) (Table 3). In the LSS group, poor sleep quality was associated with multilevel stenosis, the ODI score, ODI score without a sleep component, and EQ-5D (Table 3).

### 3.3. Correlation between Global PSQI Score and Both Disability and Quality of Life

Table 4 demonstrates the regression model for predicting VAS for back pain in the ASD group. As possible independent variables, age, sex, VAS for leg pain, and global PSQI score were entered in the model. The predictors accounted for 31.5%, which was statistically significant (*P*=0.005). The beta coefficients for PSQI and VAS for leg pain were all positively associated with VAS for back pain, suggesting that poor sleep quality and increased leg pain were associated with increased back pain (Table 4).

## 4. Discussion

The present study showed that 75% of patients with ASD had poor sleep quality, which corresponded to the prevalence of poor sleep quality in the LSS group (72.7%). Like in other studies about the relationship between pain and sleep quality [3, 11, 13, 25], poor sleep quality was associated with the VAS score for back pain in the ASD group. A regression model also supported the relationship between back pain and sleep disturbance in patients in the ASD group.

The prevalence of sleep disturbance was 75% in the ASD group, as determined using the PSQI score. This prevalence was comparable to the prevalence of sleep disturbance associated with other musculoskeletal diseases, including 39.1–56.5% in knee osteoarthritis, 71–86% in shoulder diseases, 86.6% in chronic low back pain, >90% in fibromyalgia, and 83% in degenerative spinal disease [4, 6, 7, 10, 11, 13, 20, 21]. Therefore, we believe that patients with ASD have an equally high chance of having sleep problems as patients with other musculoskeletal diseases. However, it should be acknowledged that the patients in the present study were scheduled to undergo spine surgery for ASD, and because there is a wide spectrum of symptom severity in ASD, the prevalence of sleep disturbance would depend on the symptom severity due to painful ASD.

The VAS score for back pain in patients with ASD was significantly higher in poor sleepers than in nonpoor sleepers, indicating that sleep disturbance is associated with the increase of pain perception in patients with ASD. Several studies agree with these findings, demonstrating that sleep quality is an independent predictor of disability along with pain in degenerative spinal diseases [7]. A regression model for back pain and sleep disturbance developed in this study is also in line with these results, suggesting that poor sleep quality can independently explain the increased level of back pain in patients with ASD. However, the present study was designed as a cross-sectional study. Therefore, we cannot draw a conclusion about the causal relationship between ASD and sleep disturbance. Previous studies have reported that this association between sleep and pain is cyclical and bidirectional [7, 26]. Disrupted sleep may contribute directly to increased central pain processing, thus exacerbating daily pain, which may then perpetuate sleep disturbances [27].

There was no difference in the VAS score for back pain between poor sleepers and nonpoor sleepers in the LSS group. This result might be explained by the fact that LSS causes back and leg pain primarily during walking, making claudication the pathognomonic symptom of LSS. Instead, poor sleepers in the LSS group showed significantly higher ODI scores and ODI scores without a sleep component, but lower EQ-5D than nonpoor sleepers. A previous study supports this finding [13]. Batmaz et al. reported that sleep disturbance was associated with higher levels of disability, depression, and anxiety in patients with LSS [13]. Interestingly, the use of sleep medications was significantly different among subcomponents of PSQI between the both groups. This might be because patients with LSS usually take medication such as benzodiazepine or gabapentinoid for radiculopathy, which is classified as a sedative drug or CNS depressants [28].

The present study has several limitations. First, relatively few patients were included in the ASD group. To identify the exact prevalence of poor sleep quality in patients with ASD, further studies with larger sample sizes would be necessary. In addition, this might make the differences in the VAS score for leg pain, ODI scores, and EQ-5D statistically nonsignificant. Second, the present study could not provide any information about the causal relationship between ASD and poor sleep quality. More investigations are necessary to establish whether back pain caused by ASD would be ameliorated upon improvement of sleep quality.

In conclusion, a robust relationship between ASD and sleep disturbance was observed in this study. Because sleep quality is a critical factor in augmenting back pain in patients with ASD, this study underlines the need to investigate sleep quality during the routine examination of patients with ASD.

## Figures and Tables

**Table 1:** Comparison of descriptive statistics between ASD and LSS groups.

	ASD (44)^*∗*^	LSS (137)	LSS (44)^*∗*^	*P* value^*∗*^
Age (years)	69.5 ± 7.7	68.9 ± 9.5	69.9 ± 8.0	0.804
BMI	26.5 ± 4.3	25.7 ± 3.5	25.8 ± 3.7	0.251
Female (*n* (%))	34 [77.3]	82 [59.9]	34 [77.3]	1.000
VAS for back pain	7.2 ± 2.1	6.3 ± 2.7	6.6 ± 2.6	0.238
VAS for leg pain	6.2 ± 2.9	7.1 ± 2.3	7.0 ± 2.1	0.191
ODI	48.0 ± 17.1	46.4 ± 15.2	46.5 ± 14.8	0.673
ODI without sleep component	45.0 ± 15.3	43.9 ± 14.1	44.1 ± 13.7	0.765
EQ-5D	0.319 ± 0.275	0.297 ± 0.265	0.261 ± 0.259	0.312
Coffee or tea intake (cups/day)	1.5 ± 1.1	1.6 ± 0.9	1.6 ± 1.5	0.941
Global PSQI score (0–21)	8.4 ± 4.1	8.26 ± 4.7	9.3 ± 4.8 T	0.375
Poor sleeper (*n* (%))	33 [75.0]	96 [70.1]	32 [72.7]	0.858

SD, standard deviation; ASD, adult spinal deformity; LSS, lumbar spinal stenosis; BMI, body mass index; VAS, visual analog pain scale; ODI, Oswestry Disability Index, EQ-5D, EuroQol; PSQI, Pittsburgh Sleep Quality Index; poor sleeper was defined as > 5 of the global PSQI score. ^*∗*^Comparison of values between both groups after propensity score matching. Values are presented as *n* or mean ± SD.

**Table 2:** Comparison of subcomponent PSQI scores.

	ASD (44)	LSS (44)	*P* value
Sleep quality (0–3)	1.5 ± 0.7	1.6 ± 0.7	0.418
Sleep latency (0–3)	1.6 ± 1.1	1.8 ± 1.1	0.217
Sleep duration (0–3)	1.4 ± 1.2	1.2 ± 1.2	0.423
Habitual sleep efficiency (0–3)	0.9 ± 1.0	0.8 ± 1.2	0.764
Sleep disturbance (0–3)	1.8 ± 0.6	1.8 ± 0.7	1.000
Use of sleep medications (0–3)	0.5 ± 1.0	1.1 ± 1.4	0.034
Daytime dysfunction (0–3)	0.8 ± 0.9	1.0 ± 1.0	0.320

SD, standard deviation; ASD, adult spinal deformity; LSS, lumbar spinal stenosis. Values are presented as *n* or mean ± SD.

**Table 3:** Comparison of sleep disturbance and clinical outcomes between surgical and nonsurgical treatments groups.

	ASD (44)			LSS (137)		
	Poor sleepers (33)	Nonpoor sleepers (11)	*P* value	Poor sleepers (96)	Nonpoor sleepers (41)	*P* value
Age (years)	69.8 ± 7.6	68.7 ± 8.5	0.710	68.0 ± 9.9	71.1 ± 8.3	0.085
Female (n (%))	27 [81.8]	7 [63.6]	0.145	61 [63.5]	21 [51.2]	0.178
Multilevel stenosis (n (%))	N/A	N/A		36 [37.5]	8 [19.5]	0.039
VAS for back pain	7.7 ± 1.7	5.6 ± 2.2	**0.002 (0.004)**	6.6 ± 2.4	5.6 ± 3.2	0.064 (0.079)
VAS for leg pain	6.3 ± 2.8	6.0 ± 3.4	0.765 (0.508)	7.2 ± 2.0	6.7 ± 2.8	0.245 (0.170)
ODI	50.0 ± 16.1	42.0 ± 19.4	0.185 (0.214)	48.5 ± 14.4	41.6 ± 15.9	**0.014 (0.011)**
ODI without sleep component	46.8 ± 14.2	39.8 ± 17.7	0.193 (0.221)	45.3 ± 13.4	40.4 ± 15.2	**0.063 (0.049)**
EQ-5D	0.301 ± 0.269	0.371 ± 0.301	0.475 (0.406)	0.262 ± 0.261	0.380 ± 0.259	**0.016 (0.013)**
Global PSQI score (0–21)	10.0 ± 3.5	3.8 ± 1.2	**< 0.001**	10.5 ± 3.6	3.0 ± 1.5	**< 0.001**

SD, standard deviation; BMI, body mass index; N/A, not applicable; VAS, visual analog pain scale; ODI, Oswestry Disability Index, EQ-5D, EuroQol; PSQI, Pittsburgh Sleep Quality Index. Parenthesis, the value adjusted by sex and age using analysis covariance (ANCOVA). ^*∗*^Poor sleeper was defined as >5 of the global PSQI score. Values are presented as mean ± SD.

**Table 4:** Multiple regression analysis for correlation between back pain and sleep disturbance in the ASD group.

Dependent variable	Model (independent variable)	*r* ^*2*^	*β*	*P* value
VAS for back pain		**0.315**		**0.005**
	PSQI		0.310	**0.041**
	Age		−0.023	0.867
	Sex		0.102	0.494
	VAS for leg pain		0.411	**0.007**

VAS, visual analog pain scale; ODI, Oswestry Disability Index, EQ-5D, EuroQol; PSQI, Pittsburgh Sleep Quality Index.

## Data Availability

The data used to support the findings of this study are included within the article.
